# Effect of X-ray irradiation on development, flight, and reproduction of *Spodoptera litura*

**DOI:** 10.3389/fphys.2022.947848

**Published:** 2022-07-18

**Authors:** Shan Jiang, Xiao-Wei Fu, Shan-Shan Jiang, Xian-Ming Yang, Hui-Yuan Zhao, Kongming Wu

**Affiliations:** ^1^ College of Plant Protection, Shenyang Agricultural University, Shenyang, China; ^2^ State Key Laboratory for Biology of Plant Diseases and Insect Pests, Institute of Plant Protection, Chinese Academy of Agricultural Sciences, Beijing, China; ^3^ Henan Institute of Science and Technology, Xinxiang, China; ^4^ Hebi Jiaduo Industry and Trade Co., Ltd., Hebi, China

**Keywords:** *Spodoptera litura*, sterile insect technology, X-ray, radiation biology, release ratios

## Abstract

*Spodoptera litura* is an omnivorous pest that has spread globally. Because irradiation sterilization technology has a great potential for control of *S. litura*, the effect of 25–150 Gy doses of X-rays on pupal survival, flight and reproductive variables of adult moths were analyzed in this research. The X-ray irradiation with the dose of 25–150 Gy significantly affected the reproductive ability of females. Irradiating male pupae with 25–150 Gy doses of X-rays had no effect on mating, life span, or flight ability of adult moths, but significantly reduced survival and fecundity of their offspring, and the sterility rate of the F_1_ generation was 52.65%–99.9%. The results of logistic curve fitting showed that the sterility impact was 84% at the most appropriate irradiation dose (71.26 Gy). The sterility control was 91% in an indoor mating competition experiment when the release ratio of irradiated males (75 Gy) to nonirradiated males reached 12.6:1. The effects of X-ray irradiation doses on biological variables of *S. litura* and the most effective release ratio determined here provide a theoretical foundation for using radiation sterilization technology to control *S. litura*.

## 1 Introduction

*Spodoptera litura* (Fabricius) (Noctuidae; Lepidoptera) is a worldwide pest (Bishara, 1934; Aitkenhead et al., 1974; Tojo et al., 2013) that can feed on 389 plant species from 109 families (Qin et al., 2006), it is also widely dispersed in China, where it breeds all year in Yunnan and other tropical areas of China (Wu et al., 2016). Chemical pesticides are the most common way to control *S. litura*, but resistance to a range of chemical insecticides (Srivastava and Joshi, 1965; Zhou and Huang, 2002; Ahmad et al., 2008), particularly carbamates and pyrethroids, has been reported since 1965 (Zhou and Huang, 2002; Ahmad et al., 2007a; Ahmad et al., 2007b). As a result, strategies for integrated pest management (IPM) of *S. litura* have been widely accepted by all stakeholders (Rao et al., 1993; Klassen, 2005; Ehler, 2006).

One measure for IPM is sterilizing insect pests by irradiation with X-rays, electron beams, or γ-rays, then releasing a large number of sterile insects into the field (Knipling, 1955; LaChance et al., 1975). Since the 1950s, this eco-friendly technology has been used in wide range of IPM applications to control pests (Hendrichs et al., 2002) such as the dipterans *Cochliomyia hominivorax* (Coquerel) (Knipling, 1960; Wyss, 2006), *Ceratitis capitata* (Wiedemann) (De Longo et al.,2000; Hendrichs et al., 1983), *Bactrocera cucurbitae* (Coquillett) (Yosiaki et al., 2003; Koyama et al., 2004). The use of radiation sterilization has lagged for lepidopterans, however, because of their high resistance to radiation (LaChance, 1967). As a result, a method to generate inherited sterility (IS) was developed for lepidopteran insects (Proverbs and Newton, 1962; North, 1975) and has been used to control a group of lepidopteran insects such as *Cydia pomonella* (Bloem et al., 2007), *Teia anartoides* (Walker) (Suckling et al., 2007), *Pectinophora gossypiella* (Saunders) (Bloem et al., 2005; Tabashnik et al., 2021). However, because radioisotopes cannot be guaranteed to be safe, their usage is becoming increasingly limited. Many SIT programs, which rely on ionizing radiation from radioisotopes for insect sterilization, are severely hampered. As a result, finding a replacement for radioisotopes radiation source is critical. Because of its higher safety, lack of radioactive residue, and ease of portability, X-rays have steadily replaced highly radioactive cobalt sources that were commonly used in the past (Bakri et al., 2005; Yamada et al., 2014; Light et al., 2015).

The method of employing X-rays to irradiate insects has gradually gained traction in recent years, and considerable research has been reported. Cagnotti et al. (2012) used X-rays to sterilize *Tuta absoluta* pupae and found that 200 Gy was the best dose for sterility. Urquidi et al. (2015) used different wavelengths of X-rays to irradiate mosquitos, finding that long wavelengths were more effective at mosquito sterilization. Adult orangeworm moths were irradiated with X-rays by Light et al. (2015), the research showed that 125 Gy could cause sterility in both parents and F_1_ generations. *Aedes albopictus* irradiated with X-ray at a dose of 40 Gy by Du et al. (2019) also had a high sterility effect. Haff et al. (2020) discovered that X-rays and γ-rays were biologically equivalent after irradiating *Amyelois transitella* larvae with X-rays. With 200 Gy X-ray treatment, *Ephestia elutella* can be sterilized, and a 15:1 ratio of irradiated male insects to normal male insects can prevent 71.91% of the wild population from reproducing (Zhao et al., 2022). In 1974, scientists discovered that 40 Gy of ^60^Co radiation had a significant impact on the offspring development of male *S. litura* (Mochida and Miyahara, 1974). In 1993, Seth and Sehgal explored the influence of radiation on the longevity of male *S. litura* and its F_1_ generation larvae, proving that IS may be used to manage *S. litura* (Seth and Sehgal, 1993). However, no any study on the optimal irradiation dose of X-rays to control *S.litura* was reported until now. In this study, we determined the effects of X-ray irradiation doses on critical biological variables and the best release ratio of irradiated to nonirradiated males to provide a theoretical basis for the development of *S. litura* green control technology.

## 2 Materials and methods

### 2.1 Experimental insect

Adults of *S. litura* were obtained from the Langfang Experimental Station, Institute of Plant Protection, Chinese Academy of Agricultural Sciences, where field populations were collected on a regular basis for rejuvenation. After eclosion, adults were placed in a 450 ml disposable plastic bottle with a cotton ball dipped in 5% v/v honey water, and then the bottle was sealed with gauze. Daily, any eggs in the bottle were removed and placed in a self-sealed bag to await hatching, and the cotton ball was replaced with a fresh one. After eggs hatched, each larva was placed in a separate transparent plastic box (diameter: 50 mm; height: 40 mm) and an artificial feed made of soybean powder and wheat germ powder (Greene et al., 1976). On the fifth day after pupation, males and females were identified as described by Zhao et al. (2011). All insect stages were grown in an MGC intelligent program artificial climate box (MGC-450HP, Shanghai Yiheng Scientific Instrument Co., Ltd.) at 25°C ± 1°C, 70% ± 5% RH, and photoperiod of L: D = 16 h: 8 h.

### 2.2 Irradiation equipment

An X-ray irradiator (JYK-001 type), newly developed by Hebi Jiaduoke Industry and Trade Co., Ltd., Hebi, China, was used. The dose of X-ray was selected as 1.790 mGy/s (180 kV/10 mA) in this study. The samples were placed on an irradiation table (210 mm × 210 mm) at a distance of 400 mm from the X-ray source to receive X-rays. The temperature in the irradiation chamber was 25°C ± 1°C, and the irradiation gradient was 0 Gy (unirradiated), 25 Gy (13,416 s), 50 Gy (26,831 s), 75 Gy (40,247 s), 100 Gy (53,662 s), 125 Gy (67,078 s), and 150 Gy (80,494 s). When the cumulative dose was attained, the irradiation was halted and the sample was taken out. During irradiation, the dosage rate was monitored in real-time and ranged from 1.731 to 1.864 mGy/s.

### 2.3 Experimental methods

#### 2.3.1 Effects of irradiation on *S. litura* pupa

Healthy male and female pupae with a pupal mass >300 mg near eclosion (8 days old) (Bushland and Hopkins, 1951; Ouye et al., 1964) were placed in different transparent boxes (length: 100 mm; width: 100 mm; height: 10 mm) according to the irradiation dose with wet gauze to maintain humidity. 30 female and 30 male pupae were irradiated for each of the six doses (25 Gy, 50 Gy, 75 Gy, 100 Gy, 125 Gy, 150 Gy), using unirradiated insects cultured in the lab at the same time as the control. The experiment was repeated six times. Each pupa was then placed in a 450 ml bottle to wait for eclosion after being irradiated. Dead and deformed pupae were used to calculate mortality and deformity rates; pupae were considered deformed if moths could not expand their wings after eclosion.

#### 2.3.2 Effects of X-ray irradiation on mating and reproduction of the parent moths of *S. litura*

Pupae were irradiated as described in Section 2.3.1. After the adult emerged, they were paired with unirradiated females; the pair was placed in a 450 ml plastic bottle, eggs collected and food replaced as described in Section 2.1. The pairing methods of moths are as follows: N♀ × N♂ (control), N♀ × T♂, and T♀ × N♂ (N, nonirradiated, T, irradiation treatment); 15 pairs were treated per dose, and the experiment was done 4 times. The spermatophores within the spermathecae were counted and the mating history of the dead female moths was determined using a stereoscope with 1× objective (TS-63X, Shanghai Shangguang New Optical Technology Co., Ltd., Shanghai, China). When spermatophore was found to exist in the female moth’s spermathecae, the moth mating was identified successfully. Pre-oviposition, oviposition, and post-oviposition periods (the time between the last egg laying and the female’s death), the number of eggs deposited, eggs hatched, male adult lifespan, and mating time for each group were all recorded. Finally, the infertility rate was calculated (the probability that the eggs laid by female moth did not hatch was referred as the infertility rate).

#### 2.3.3 Effects of different X-ray irradiation doses on flight ability of *S. litura* adults

Flying ability was assessed using the FXMD-24-USB insect flight information system (Henan Hebi Jiaduoke Industry and Trade Co., Ltd.) and the hoisting test based on the method of Ge et al. (2019). Male pupae were irradiated as described in Section 2.3.1. After emergence of the adults, 3-day-old adults that developed from the irradiated pupae and had intact wings were loaded into the finger tube and numbered. Twenty to thirty adults were selected from each treatment dose. The moths were removed carefully from the tubes, the wings of the moth were stretched out, the scales on the abdomen and thorax were brushed away, and the thorax was attached to the flight mill with a small droplet of cyanoacrylate glue (Deli Group Co., Ltd., Zhejiang, China). The test insect was inserted vertically at 90 degrees onto the crane arm of the flight mill and allowed to fly continuously for 24 h in the dark at 25 C ± 1°C and 70% ± 5% RH.

#### 2.3.4 Effects of different X-ray irradiation on F_1_ generations of *S. litura*

F_1_ generation larvae (the male parent was irradiated [see Section 2.3.1], the female parent was not) were provided artificial feed (see Section 2.1 for insect rearing methods), their development was observed every day, and the duration of each stage was recorded. Also, the 3-days-old pupae were weighed. Once the F_1_ adults had emerged, they were paired with nonirradiated heterosexual adults that emerged on the same day as F_1_ adults, and reproductive variables (such as the periods of pre-oviposition, oviposition, and post-oviposition; the number of eggs deposited, eggs hatched, adult lifespan, and mating rate) were recorded. Growth of at least 200 eggs and 80 first-instar larvae were observed for each treatment, and the experiment was repeated three times. Mating rate and sterility rate were calculated for the F_1_ generation. Finally, the life table parameters for the F_1_ population were calculated. The net reproductive rate (*R*_0_), generation time (*T*), intrinsic rate of increase (*r*), and finite rate of increase (*λ*) of the *S.litura* F_1_ populations were estimated using the formulas below (Chi and Liu, 1985; Chi 1988; Asiimwe et al., 2007):

$$\text{λ}=e^{r}$$

$$R_{0}=\overset{\infty }{\underset{x=0}{\sum }}l_{x}m_{x}$$

$$T=\frac{\ln R_{0}}{r}$$

$$l_{x}=\overset{m}{\underset{j=1}{\sum }}S_{xj}$$

$$m_{x}=\frac{\sum_{j=1}^{m}S_{xj}f_{xj}}{\sum_{j=1}^{m}S_{xj}}$$

$$\overset{\infty }{\underset{x=0}{\sum }}l_{x}m_{x}e^{-r\left(x+1\right)}=1\;$$

The *x* is the number of days, *l*_*x*_ is the *S. litura* survival probability from egg to *x* days old *f*_*x*_ is the age-specific fecundity at age *x*, *m*_*x*_ is the average population fecundity from egg to *x* days old, *l*_*x*_ *m*_*x*_ is the population age-specific maternity, and *e* is the Euler number.

#### 2.3.5 Mating competition test between irradiated and nonirradiated males of *S. litura*

We set up a total of 13 groups with differing numbers of irradiated males (75 Gy X-rays in the pupal stage) to nonirradiated males to nonirradiated normal females: 0:1:1 (control), 1:1:1, 2:1:1, 3:1:1, 4:1:1, 5:1:1, 6:1:1, 7:1:1, 8:1:1, 9:1:1, 10:1:1, 11:1:1, and 12:1:1 in feeding buckets (diameter: 25 cm; height: 30 cm) with cotton balls dipped in 5% v/v honey water. The pail was sealed with gauze, eggs were collected and counted and fresh food supplied daily. The number of eggs which hatched larva was also recorded for calculating the infertility rate.

### 2.4 Data analyses

A two-way analysis of variance was used to examine the deformity rate and adjusted mortality of irradiated pupae, and the reproductive parameters of parental adult moths, with different radiation doses and sex as factors. If the difference was significant, Tukey’s HSD multiple comparison test was performed. The one-way ANOVA was used to analyze the effects of different irradiation doses on mean adult flight ability, and F_1_ insect development of *S. litura* in software SPSS (version 26.0; IBM, Armonk, NY, United States). Percentage data were arcsine square-root transformed before the analysis of variance. If the difference among doses was significant for a variable, multiple Tukey’s HSD comparisons were carried out, and the Log-rank test was used to analyze the survival of the F_1_ generation of *S. litura* with different dose treatments in GraphPad (version 8.0; GraphPad Software Inc., San Diego, CA, United States).

Logistic regression was used to fit the sterility rate of *S. litura* at different doses of X-rays, and the sterility rate of *S. litura* with different release ratios was fitted by logistic in Origin software (version 2019; Origin Lab Corporation, Northampton, MA, United States). The model equation was $y=\frac{k}{1\;+\;e^{a\;-\;rx}}$ , where *y* is the sterility rate, *k* is the theoretical highest percentage, *x* is irradiation dose or irradiation insect release ratio, *r* is the growth rate coefficient, *a* is a shape parameter. All the figures were drawn using Graphpad 8.0. The experimental population’s life table parameters were calculated using the software TWOSEX-MSChart (Chi, 2009; Chi et al., 2020). Life table parameters were analyzed for significant differences among different treatments using a paired bootstrap test (*n* = 100,000) (Akca et al., 2015; Wei et al., 2020).

## 3 Results

### 3.1 Effect of irradiation on pupae

The deformity rate of *S. litura* pupae did not differ significantly by irradiation doses and irradiation dose × sex (irradiation doses: *F*_6, 84_ = 0.539, *p* = 0.777; irradiation dose × sex: *F*_6, 84_ = 0.790, *p* = 0.581), but did significantly by sex (*F*_1, 84_ = 7.424, *p* = 0.008), according to two-way ANOVA (Table 1). The adjusted mortality rate of *S. litura* pupae did not differ significantly by irradiation doses, sex, and irradiation dose × sex (irradiation doses: *F*_6, 72_ = 0.361, *p* = 0.900; sex: *F*_1, 72_ = 0.181, *p* = 0.672; irradiation dose × sex: *F*_6, 72_ = 0.168, *p* = 0.984).

**TABLE 1:** Mean (±SE) deformity rates and adjusted mortality of pupae of *Spodoptera litura* after irradiation with different doses of X-rays.

Irradiation dose (Gy)	Male		Female	
	Deformity rate (%)	Adjusted mortality (%)	Deformity rate (%)	Adjusted mortality (%)
0 (control)	11.65 ± 2.02a	—	16.82 ± 3.39a	—
25	13.81 ± 4.22a	0.02 ± 0.06a	25.40 ± 4.22a	−0.05 ± 0.03a
50	19.21 ± 2.73a	0.07 ± 0.05a	16.73 ± 1.83a	0.10 ± 0.06 a
75	14.06 ± 3.01a	0.08 ± 0.10a	20.23 ± 3.88a	0.09 ± 0.08a
100	13.46 ± 2.51a	0.18 ± 0.10a	21.07 ± 2.53a	0.00 ± 0.05a
125	13.91 ± 4.22a	0.12 ± 0.11a	18.8 ± 5.67a	0.14 ± 0.08a
150	13.82 ± 3.62a	0.09 ± 0.10a	16.25 ± 2.59a	0.01 ± 0.03a

Note: Among different irradiation doses, significant differences in pupal deformity and adjusted mortality of *S. litura* (two-way ANOVA, Tukey’s HSD; *p* < 0.05) are shown by different lowercase letters.

### 3.2 *S. litura* reproductive parameters and adult longevity after various X-ray doses

**FIGURE 1:**
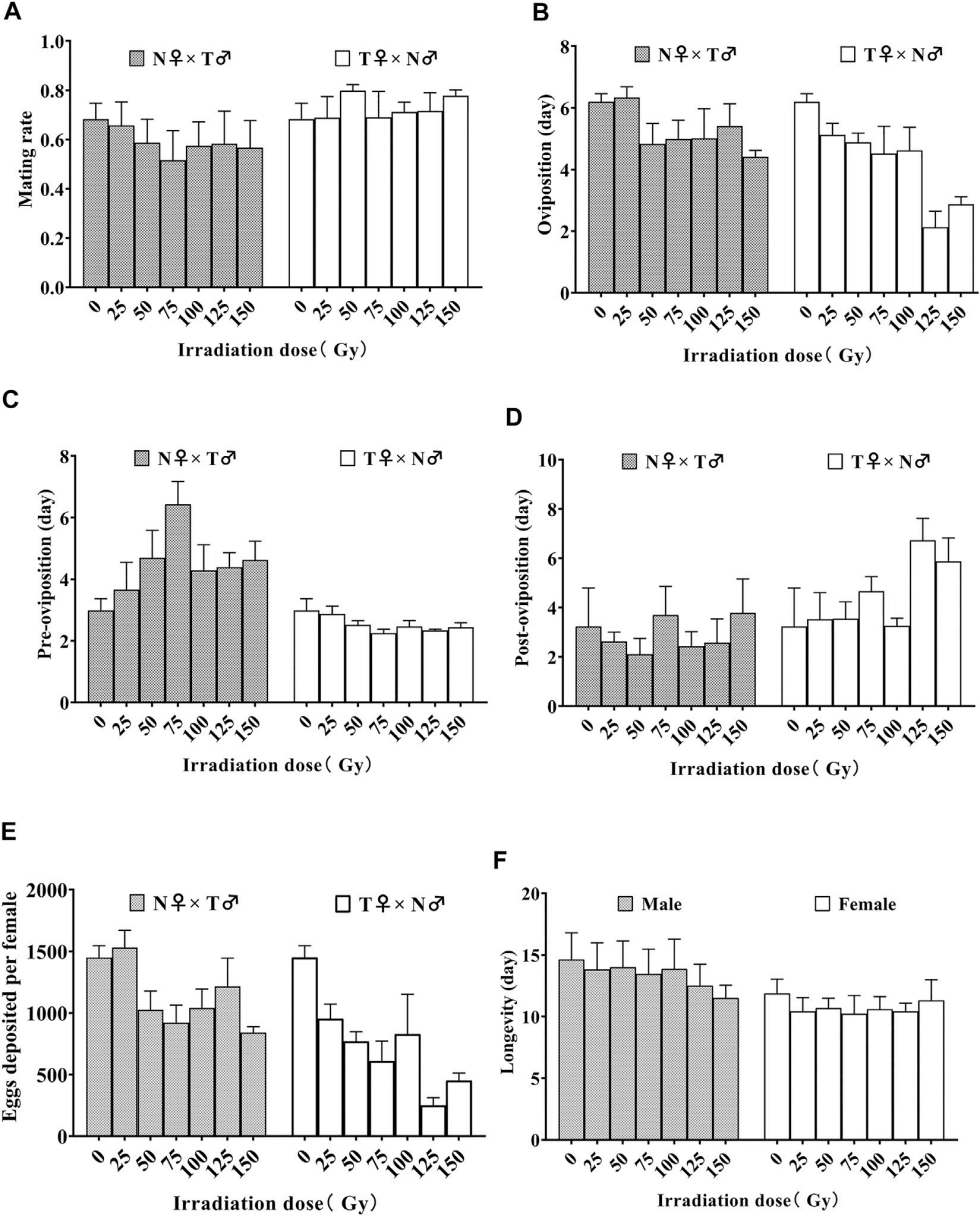
Effect of different doses of X-rays on mean (±SE) mating rate **(A)**, -oviposition **(B)**, pre-oviposition **(C)**, post-oviposition **(D)**, eggs deposited per female **(E)**, and the longevity of irradiated moth **(F)** adults of patent *Spodoptera litura*. The pairings were N♀ × T♂ (N, no irradiation; T, irradiation treatment) while the control pairings were N♀ × N♂ (0 Gy).

The mating rate of *S. litura* parents was not affected by irradiation doses and irradiation dose × sex interaction, but it did significantly by sex (irradiation doses: *F*_6, 42_ = 0.241, *p* = 0.9602; sex: *F*_1, 42_ = 7.574, *p* = 0.009; irradiation dose × sex: *F*_6, 42_ = 0.459, *p* = 0.834; Figure 1A). The oviposition of *S. litura* parents was affected by irradiation dose and sex, but not by irradiation dose × sex interaction (irradiation doses: *F*_6, 42_ = 5.469, *p* = 0.0003; sex: *F*_1, 42_ = 10.460, *p* = 0.002; irradiation dose × sex: *F*_6, 42_ = 2.180, *p* = 0.064; Figure 1B). The irradiation dose × sex interaction had a significant influence on the pre-oviposition duration, while the irradiation dose had no effect (irradiation doses: *F*_6, 42_ = 1.292, *p* = 0.282; sex: *F*_1, 42_ = 45.30, *p* < 0.0001; irradiation dose × sex: *F*_6, 42_ = 3.106, *p* = 0.013; Figure 1C). The sex had a significant effect on the post-oviposition duration, while the irradiation dose and irradiation dose × sex interaction had no effect (irradiation doses: *F*_6, 42_ = 1.525, *p* = 0.194; sex: *F*_1, 42_ = 7.796, *p* = 0.008; irradiation dose × sex: *F*_6, 42_ = 0.909, *p* = 0.498; Figure 1D). The eggs deposited per female was affected significantly by irradiation dose and sex, but not by the interaction of irradiation dose and sex (irradiation doses: *F*_6, 42_ = 7.519, *p* < 0.0001; sex: *F*_1, 42_ = 23.250, *p* < 0.0001; irradiation dose × sex: *F*_6, 42_ = 2.113, *p* = 0.0718; Figure 1E). The longevity of *S. litura* adults was affected significantly by sex, but not by irradiation dose and irradiation dose × sex interaction (irradiation doses: *F*_6, 42_ = 0.2928, *p* = 0.9370; sex: *F*_1, 42_ = 8.746, *p* = 0.0051; irradiation dose × sex: *F*_6, 42_ = 0.2499, *p* = 0.9566; Figure 1F).

### 3.3 Effects of different doses on sterility of *S. litura*

**FIGURE 2:**
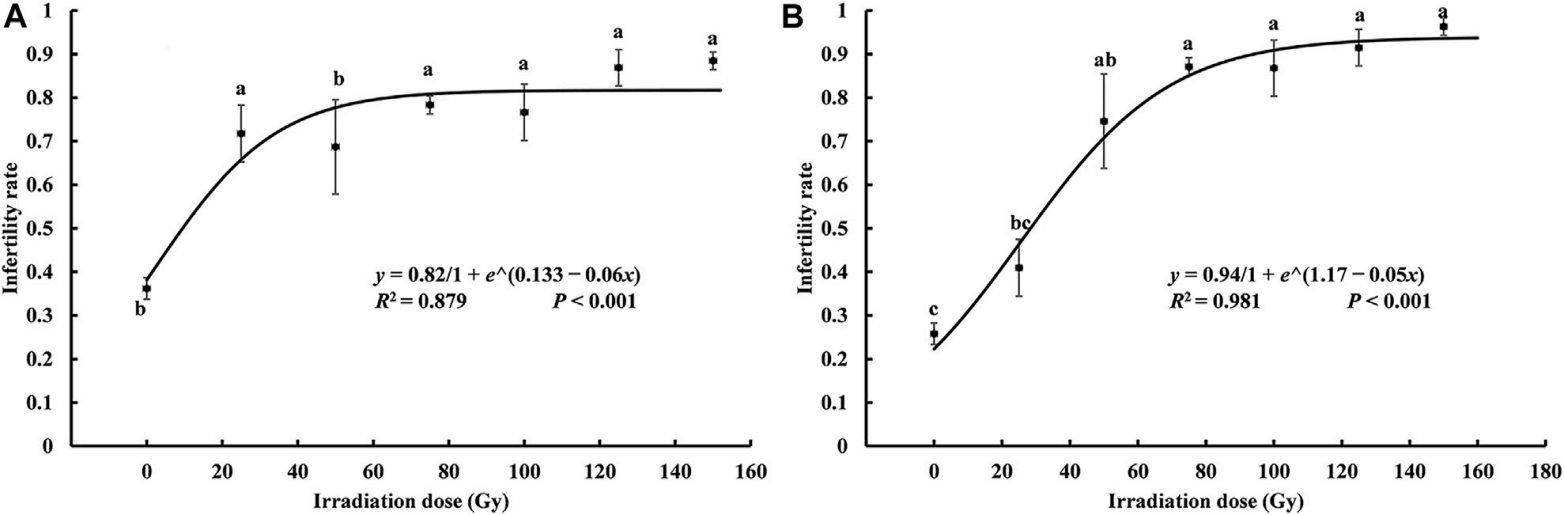
Curve of the relationship between the sterility rate of *Spodoptera litura* hybrids and X-ray irradiation dose. **(A)** the sterility rate of X-ray irradiated female paired with unirradiated male; **(B)** the sterility rate of unirradiated female paired with of X-ray irradiated male. Data are means ± SE; significant differences (one-way ANOVA, Tukey’s HSD; *p* < 0.05) among doses for a variable are indicated by different lowercase letters.

According to a one-way ANOVA, the sterility rate for irradiated moths that mated with nonirradiated heterosexual moths increased significantly at higher irradiation doses (T♀ × N♂: *F*_6, 21_ = 5.514, *p* = 0.001; N♀ × T♂: *F*_6, 21_ = 13.611, *p* < 0.001) (N = nonirradiated, T = irradiation treatment), and both the growth dynamics fit the logistic curve (T♀ × N♂: *R* ^*2*^ = 0.879, *p* < 0.001; N♀ × T♂: *R* ^*2*^ = 0.981, *p* < 0.001, Figure 2). The equation that fits the mating sterility rate of irradiated male moths and nonirradiated female moths was $y=\frac{0.82}{1\;+\;e^{0.133\;-\;0.06x}}$ . The endpoint of the rapid growth of the sterility rate was calculated to be 59.17 Gy based on the inflection point of the logistic curve (Zhang and Liu, 1992). The equation that fit the mating sterility rate of irradiated male moths and nonirradiated female moths was $y=\frac{0.94}{1\;+\;e^{1.17\;-\;0.05x}}$ (Figure 2). The endpoint of the rapid growth of the sterility rate was calculated to be 71.26 Gy based on the inflection point of the logistic curve. That is, when the irradiation dose was less than 71.26 Gy, the sterility rate of the irradiated male pupae mating with normal adults after eclosion all had a rapid growth trend, but when the dose was greater than 71.26 Gy, the rate of infertility tends to level off. In the theory, the appropriate X-ray dose for male *S. litura* pupae should be ≥71.26 Gy for obtaining a better control effect.

### 3.4 Effects of X-ray irradiation on the flight ability of *S. litura*

In the one-way analysis of variance, none of the flight variables differed significantly among the irradiation doses (flight mass loss: *F*_6, 125_ = 0.241, *p* = 0.962; flight speed: *F*_6, 125_ = 1.147, *p* = 0.339; flight time: *F*_6, 125_ = 0.963, *p* = 0.453; flight distance: *F*_6, 125_ = 0.992, *p* = 0.434) (Table 2).

**TABLE 2:** Means (±SE) for flight performance variables of *Spodoptera litura* adults exposed to different doses of X-ray during pupal stage.

Irradiation dose (Gy)	Mass loss (mg)	Velocity (km/h)	Duration (h)	Distance (km)
0 (control)	56.87 ± 5.44a	2.05 ± 0.25a	8.56 ± 1.62a	19.51 ± 4.41a
25	51.33 ± 1.92a	1.56 ± 0.15a	6.15 ± 1.57a	10.40 ± 2.85a
50	51.48 ± 4.00a	1.90 ± 0.26a	10.25 ± 1.99a	21.98 ± 5.64a
75	52.62 ± 4.29a	1.73 ± 0.20a	7.45 ± 1.95a	14.84 ± 6.28a
100	47.36 ± 4.89a	1.31 ± 0.20a	3.09 ± 0.80a	3.90 ± 0.86a
125	54.58 ± 3.48a	1.96 ± 0.26a	4.56 ± 1.94a	8.99 ± 3.80a
150	58.70 ± 2.20a	2.19 ± 0.46a	7.60 ± 1.29a	18.32 ± 5.32a

Note: Among different irradiation doses, significant differences in flight performance of *S. litura* (one-way ANOVA, Tukey’s HSD; *p* < 0.05) are shown by different lowercase letters.

### 3.5 Development of *S. litura* F_1_ generations after males were irradiated with different doses of X-rays

After males that were irradiated at doses from 25 to 150 Gy at the pupal stage mated with nonirradiated females, the developmental duration of the F_1_ generation insects was significantly affected. As shown in the ANOVA, different doses of irradiation had significant effects on the duration of egg stage (*F*_6, 2858_ = 87.842, *p* < 0.001), 1st instar larva (*F*_6, 2833_ = 94.382, *p* < 0.001), 2nd instar larva (*F*_6, 2803_ = 29.079, *p* < 0.001), 3rd instar larva (*F*_6, 2765_ = 6.434, *p* < 0.001), 4th instar larva (*F*_6, 2702_ = 18.472, *p* < 0.001), 5th instar larva (*F*_6, 2655_ = 9.166, *p* < 0.001), 6th instar larva (*F*_6, 2440_ = 5.362, *p* < 0.001), larval stage (*F*_6, 2858_ = 4.255, *p* < 0.001), pupal stage (*F*_6, 1278_ = 5.941, *p* < 0.001), egg stage-pupal stage (*F*_6, 2858_ = 13.54, *p* = 0.03), pupal mass (*F*_6, 1278_ = 19.856, *p* < 0.001), adult duration (*F*_6, 1278_ = 3.283, *p* = 0.003), and generation period (*F*_6, 2858_ = 14.016, *p* < 0.001), the duration of pre-oviposition stage of females (*F*_6, 261_ = 3.921, *p* = 0.001), number of eggs deposited per female (*F*_6, 261_ = 4.522, *p* < 0.001) of insects, but had no significant effect on the duration of post-oviposition stage (*F*_6, 261_ = 1.304, *p* = 0.255) (Table 3). Compared with the control, the pupal mass of the F_1_ generation in the 25–150 Gy irradiation treatments group was significantly reduced (386.45–421.76 mg), and the sex ratio was significantly inclined to males (male: female = 1.04–2); the oviposition period (4–4.68 days) for the 50 Gy, 75 Gy, and 125 Gy treatment groups was significantly lower compared with the control (6.7 days); and the 100 Gy and 150 Gy treatment groups had significantly lower pre-oviposition (1.5–1.76 days) than the control group (3.32). Significantly fewer eggs were deposited per female (99.5–316.78) compared with the control (1028.3) when the irradiation dose was >125 Gy, and at 150 Gy, the insect generation duration (26.19 days) was significantly lower than that of the nonirradiated insects (35.01 days) (Table 3). The mating rate for the 25–150 Gy dose groups was not significantly different from that of the nonirradiated insects by different mating types (N♀ × T♂: *F*_6, 14_ = 0.166, *p* = 0.982; T♀ × N♂: *F*_6, 14_ = 0.479, *p* = 0.813; T♀ × T♂: *F*_6, 14_ = 1.311, *p* = 0.315) (N = normal individual whose parents were both unirradiated; T = F_1_ individual whose male parent was irradiated with 250 Gy dose of X-ray); however, when the irradiation dose ≥50 Gy, the sterility rate of the F_1_ generation with two mating types was significantly higher than that of the control (N♀ × T♂: *F*_6, 159_ = 8.973, *p* < 0.001; T♀ × T♂: *F*_6, 159_ = 10.238, *p* < 0.001). The sterility rate of the 100 Gy, 125 Gy, and 150 Gy treatment groups was significantly higher than that of the control group when the mating mode was T♀ × N♂ (*F*_6, 159_ = 5.654, *p* < 0.001) (Table 4).

**TABLE 3:** Mean duration (±SE) for developmental stages of F_1_ generation of *Spodoptera litura* after male parent was irradiated with different X-ray doses during pupal stage.

Developmental stage	Developmental duration (d)						
	0 Gy (control)	25 Gy	50 Gy	75 Gy	100 Gy	125 Gy	150 Gy
Egg	3.60 ± 0.02a	3.33 ± 0.03c	3.49 ± 0.03b	3.44 ± 0.02b	3.00 ± 0.00e	3.40 ± 0.03bc	3.06 ± 0.02d
1st instar larva	3.02 ± 0.02c	3.33 ± 0.04b	3.09 ± 0.03c	2.87 ± 0.03d	4.10 ± 0.06a	3.38 ± 0.05b	3.35 ± 0.06b
2nd instar larva	2.36 ± 0.03c	2.62 ± 0.04b	2.34 ± 0.04cd	2.21 ± 0.03d	2.65 ± 0.07b	2.93 ± 0.06a	2.67 ± 0.06b
3rd instar larva	2.49 ± 0.04b	2.70 ± 0.05a	2.86 ± 0.07a	2.26 ± 0.03c	2.83 ± 0.06a	2.23 ± 0.06c	2.92 ± 0.09a
4th instar larva	2.20 ± 0.03bc	2.06 ± 0.05c	2.26 ± 0.07bc	2.12 ± 0.03c	2.28 ± 0.07bc	2.79 ± 0.07a	2.42 ± 0.09b
5th instar larva	2.15 ± 0.03b	2.17 ± 0.06ab	2.09 ± 0.06b	2.31 ± 0.03a	1.83 ± 0.05c	2.06 ± 0.08bc	2.10 ± 0.08bc
6th instar larva	5.17 ± 0.04a	4.93 ± 0.07abc	5.06 ± 0.07ab	5.05 ± 0.04ab	4.89 ± 0.06bc	4.76 ± 0.07c	5.03 ± 0.09ab
Larval stage	19.90 ± 0.14ab	20.44 ± 0.17a	20.03 ± 0.20ab	19.51 ± 0.11b	19.92 ± 0.23ab	20.08 ± 0.24ab	19.21 ± 0.28b
Pupa	10.22 ± 0.09b	10.22 ± 0.08b	10.35 ± 0.10b	10.16 ± 0.07b	10.35 ± 0.09b	10.35 ± 0.16b	11.17 ± 0.16a
Egg-pupa	24.06 ± 0.26bc	27.38 ± 0.37 a	25.21 ± 0.38b	23.79 ± 0.23c	24.64 ± 0.42bc	24.43 ± 0.44bc	22.75 ± 0.48c
Pupa mass (mg)	440.38 ± 3.74a	397.77 ± 3.89c	394.12 ± 5.16c	421.76 ± 3.72b	402.54 ± 5.10bc	386.45 ± 5.12c	394.97 ± 6.26c
Adult	13.15 ± 0.38a	11.68 ± 0.51ab	11.51 ± 0.68ab	10.88 ± 0.38ab	10.98 ± 0.58b	12.55 ± 0.81b	10.82 ± 0.95b
Total generation	29.42 ± 0.51b	35.33 ± 0.75a	30.97 ± 0.76b	28.37 ± 0.44bc	29.66 ± 0.76b	29.7 ± 0.87b	26.19 ± 0.84c
Sex ratio (♂:♀)	0.97 ± 0.12b	1.04 ± 0.15 a	1.24 ± 0.21a	1.48 ± 0.17a	1.74 ± 0.32a	1.56 ± 0.33a	2.00 ± 0.57a
Oviposition period	6.70 ± 0.36a	5.69 ± 0.45ab	4.50 ± 0.69b	4.68 ± 0.38b	5.38 ± 0.49ab	4.00 ± 0.88b	6.13 ± 0.64ab
Pre-oviposition period	3.32 ± 0.27a	1.88 ± 0.47ab	2.17 ± 0.77ab	3.88 ± 0.39a	1.76 ± 0.24b	4.11 ± 0.79a	1.50 ± 0.27b
Post-oviposition	3.91 ± 0.37a	4.93 ± 0.63a	6.06 ± 1.12a	5.24 ± 0.65a	5.62 ± 0.81a	4.78 ± 1.08a	5.00 ± 1.02a
Eggs deposited per female	1,028.30 ± 80.79a	838.43 ± 116.74ab	511.94 ± 157.27ab	647.02 ± 103.67ab	529.33 ± 134.20ab	316.78 ± 130.39b	99.5 ± 40.76b

Note: Values in the same row followed by different lowercase letters indicate a significant difference among doses (one-way ANOVA, Tukey’s HSD; *p* < 0.05).

**TABLE 4:** Mean (±SE) mating rate and sterility rate for F_1_ offspring adults of *Spodoptera litura* by different mating types: T♀ × N♂, N♀ × T♂, and T♀ × T♂ (N = normal individual whose parents were both unirradiated; T = F_1_ individual whose male parent was irradiated with 250 Gy dose of X-ray).

Irradiation dose (Gy)	N♀ × T♂		T♀ × N♂		T♀ × T♂	
	Mating rate (%)	Sterility rate (%)	Mating rate (%)	Sterility rate (%)	Mating rate (%)	Sterility rate (%)
0 (control)	55.59 ± 3a	34.46 ± 6.44c	55.59 ± 3a	34.46 ± 6.44c	55.59 ± 3a	34.46 ± 6.44c
25 Gy	64.44 ± 16.02a	57.65 ± 7.28bc	73.33 ± 10.18a	52.65 ± 6.11bc	72.22 ± 4.01a	60.61 ± 7.95bc
50 Gy	53.82 ± 11.78a	72.3 ± 7.34ab	55.56 ± 19.44a	65.36 ± 7.56abc	66.67 ± 13.33a	89.19 ± 5.47ab
75 Gy	63.19 ± 3.03a	79.49 ± 5.48ab	68.89 ± 12.37a	65.69 ± 7.52abc	51.52 ± 3.03a	79.77 ± 7.16ab
100 Gy	53.33 ± 21.43a	79.8 ± 5.8ab	72.02 ± 14.59a	83.74 ± 5.85ab	77.38 ± 4.29a	81.21 ± 5.57ab
125 Gy	50.43 ± 17.09a	87.68 ± 5.53a	54.76 ± 2.38a	83.71 ± 7.03ab	46.67 ± 13.33a	99.9 ± 0.06a
150 Gy	54.17 ± 4.17a	92.09 ± 5.53a	55 ± 16.07a	94.46 ± 2.22a	53.33 ± 17.64a	98.02 ± 1.21a

Note: Values in the same column followed by different lowercase letters indicate a significantly difference among doses (one-way ANOVA, Tukey’s HSD; *p* < 0.05).

**FIGURE 3:**
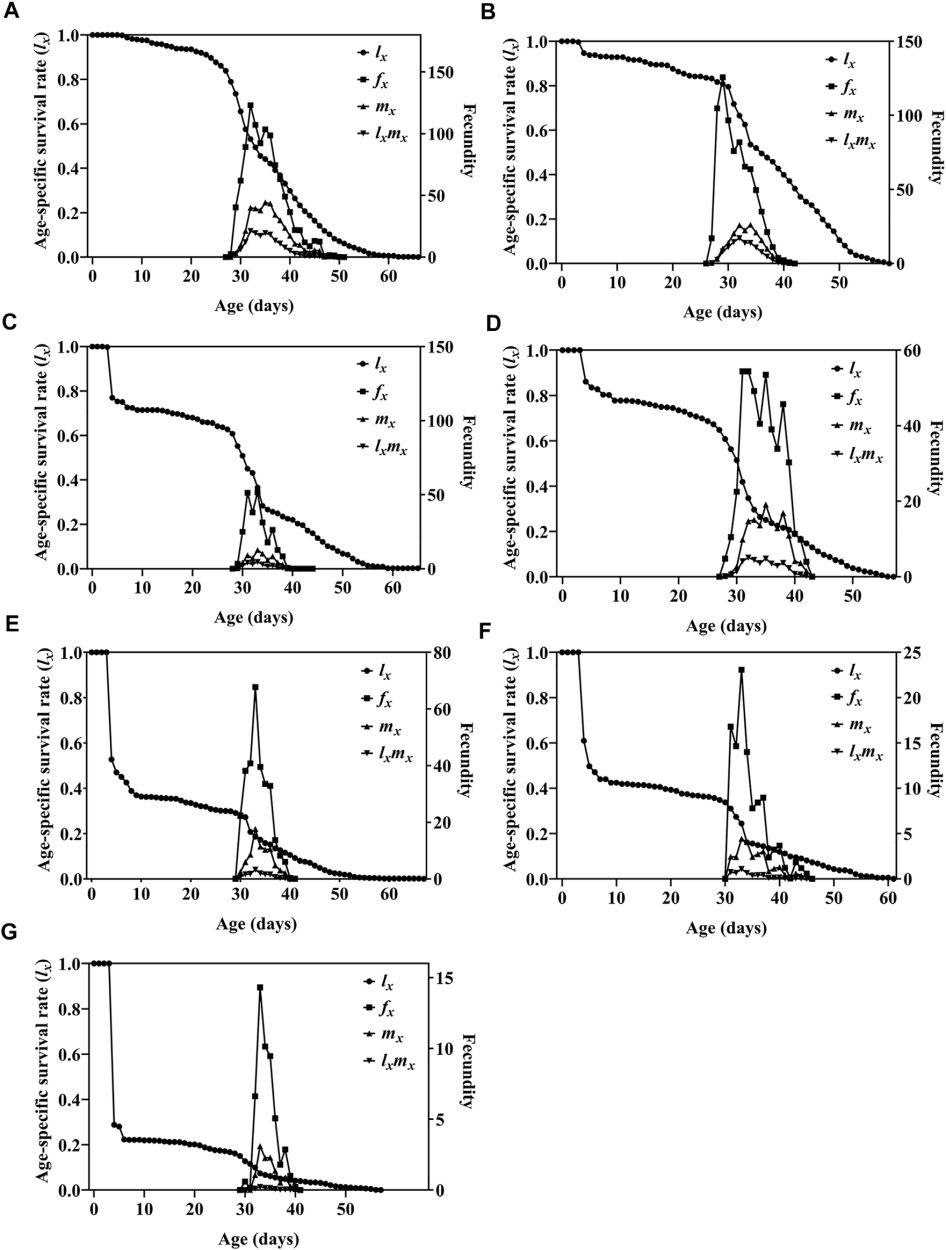
Age-specific survival rate (*l*_*x*_), age-specific fecundity of female adult (*f*_*x*_), age-specific fecundity (*m*_*x*_), and age-specific maternity (*l*_*x*_ *m*_*x*_) of F_1_ generation of *Spodoptera litura* after male parent was irradiated with different doses of X-rays. **(A)** Survival rate of F_1_ generation of nonirradiated parents (N♀ × N♂). **(B–G)** F_1_ generation after mating with nonirradiated females and males irradiated with 25 Gy, 50 Gy, 75 Gy, 100 Gy, 125 Gy, or 150 Gy doses of X-rays during pupal stage (N♀ × T♂) (N, nonirradiated; T, irradiation treatment).

Survival analysis showed that the age-specific survival rate curves (*l*_*x*_) of the population under different irradiation doses had highly significant differences (*χ* ^2^ = 904.655, *df* = 6, *p* < 0.0001), and there was a significant linear trend (*χ* ^2^ = 697.500, *df* = 1, *p* < 0.0001) (Figure 3). The *m*_*x*_ curve and the *l*_*x*_ *m*_*x*_ curve showed that the peak reproductive age of insects in the treatment group and nonirradiated insects was similar, the maximum age-specific fecundity (*m*_*x*_) for the 0–150 Gy treatment group was, respectively, on the 35th, 34th, 33rd, 35th, 33rd, 33rd, 33rd day, and the age-specific maternity (*l*_*x*_ *m*_*x*_) maximum was, respectively, on 32nd, 32nd, 33rd, 32nd, 33rd, 33rd, and 33rd day. For the 25–150 Gy doses, the age-specific fecundity of female adults (*f*_*x*_) decreased as the dose increased (highest fecundities were, respectively, 125.7, 51.6, 54.4, 67.7, 23.1, 14.3) (Figure 3). When the irradiation dose was ≥50 Gy, the net reproductive rate *R*_0_, intrinsic rate of increase *r*, and finite rate of increase *λ* of the F_1_ generation insects were significantly lower than those of the control group, and the F_1_ generation population was significantly lower (Table 5).

**TABLE 5:** Means (±SE) for life table parameters of *Spodoptera litura* F_1_ generation after male parent was irradiated with different X-ray doses during pupal stage.

Irradiation dose (Gy)	Net reproductive rate *R*_0_	Intrinsic rate of increase *r*	Mean generation time *T*	Finite rate of increase *λ*
0 (control)	163.032 ± 19.141a	0.144 ± 0.004a	35.258 ± 0.244a	1.155 ± 0.004a
25 Gy	108.943 ± 21.640a	0.140 ± 0.007a	33.304 ± 0.438c	1.150 ± 0.008a
50 Gy	22.327 ± 8.404bc	0.089 ± 0.013b	33.905 ± 0.603bc	1.093 ± 0.014bc
75 Gy	38.022 ± 7.690b	0.103 ± 0.006b	35.139 ± 0.404ab	1.108 ± 0.007b
100 Gy	13.806 ± 4.531c	0.074 ± 0.010c	34.484 ± 0.458abc	1.077 ± 0.011cd
125 Gy	4.944 ± 2.501cd	0.046 ± 0.021cd	35.431 ± 1.488abc	1.042 ± 0.021de
150 Gy	0.792 ± 0.410d	0.006 ± 0.019d	35.272 ± 1.076a	0.989 ± 0.019e

Note: Values in the same row followed by different letters differed significantly (paired bootstrap test, *p* < 0.05).

### 3.6 Competitive release ratio between irradiated males and nonirradiated males

**FIGURE 4:**
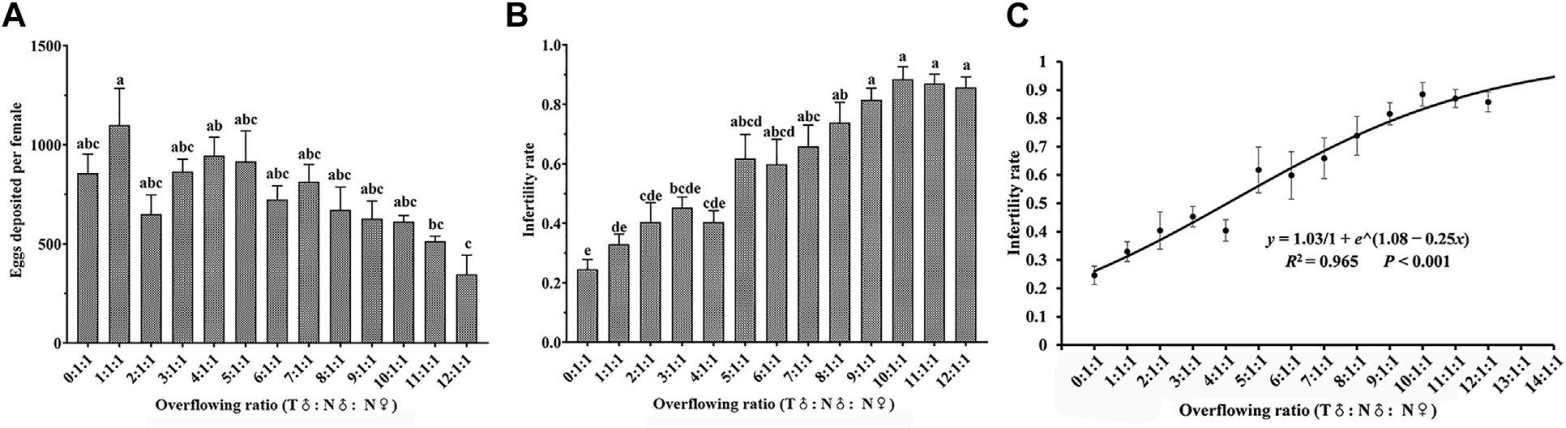
Effects of different release ratios of substerilized males (irradiated with 75 Gy dose of X-rays) and nonirradiated males on fecundity **(A)**, sterility rate **(B)** and logistic curve of sterility rate of *Spodoptera litura* **(C)**. Data are means ± SE; significant differences (one-way ANOVA, Tukey’s HSD; *p* < 0.05) among doses for a variable are shown by different lowercase letters.

There were significant differences in the number of eggs laid by females after different release ratios of irradiated males (75 Gy) and nonirradiated males (*F*_12,82_ = 2.515, *p* = 0.007, Figure 4A). The sterility rate increased significantly with an increase in proportion of irradiated males (*F*_12,82_ = 11.914, *p* < 0.001; Figure 4B). When the irradiation release ratio (T♂: N♂: N♀) was 5:1:1 to 12:1:1, the sterility rate (59.85%–88.44%) was significantly higher than in the control group (24.55%) (N = nonirradiated, T = irradiation treatment). The growth dynamics in relation to the sterility rate conformed to the logistic curve $y=\frac{1.03}{1\;+\;e^{1.08\;-\;0.25x}}$ (*x* was T♂) (*R* ^2^ = 0.965, *p* < 0.001) (Figure 4C). Thus, the turning point of the graph occurs when the radiation insect to normal insect ratio reaches 12.6:1. Theoretically, the ideal release ratio should be greater than or equal to 12.6:1:1.

## 4 Discussion

After *S. litura* pupae (8 day old) were irradiated with 25–150 Gy X-rays, our analyses of pupal survival, reproductive variables of adults, adult life span, flight ability, and offspring developmental durations showed that none of the doses altered the emergence, reproductive ability, adult life span, or flight ability of male *S. litura*. However, survival rate and fecundity of its offspring were reduced compared with nonirradiated controls, and the sex ratio of the F_1_ generation tended toward males. The X-ray of 25–150 Gy dose had no effect on the emergence rate of female pupae, but significantly affected their reproduction. However, a dose of 150 Gy still did not reach the point of female sterilization. According to the logistic curve fit, the appropriate sterilizing effect would be achieved with 71.26 Gy in theory, and the best control effect could be achieved with a release ratio of 12.6: 1 irradiated (75 Gy) males to nonirradiated males.

The type of irradiation source and the age of insects can affect the sterilization dose of insects, according to the studies on the irradiation sterility of a range of species (IDIDAS Database: https://nucleus.iaea.org/sites/naipc/ididas/Pages/Browse-IDIDAS.aspx). With a similarity, the research on *S. litura* also presented this point. In 1974, researchers discovered that exposing 3–5-day-old male pupae of *S. litura* to an 80 Gy dose of ^60^Co-rays sterilized all pupae (Mochida and Miyahara, 1974). When 3-day-old *S. litura* pupae were irradiated with a 150 Gy electron beam, the hatching rate of the eggs produced by their offspring was 0% (Yun et al., 2014). Evaluating the radiation biological characteristics of insects while using different radiation sources than in prior studies is critical. In addition, when young pupae were used, the impact of insect sterility could be achieved at a lower dose, but pupae mortality was considerable. As a result, using older pupae for irradiation would be a more cost-effective way to increase insect pupae survival after irradiation (Bushland and Hopkins, 1951; Ouye et al., 1964; Ducoff and Bosma, 1966). Among the numerous studies on irradiation-induced sterility in *S. litura*, few have focused on the effect of X-rays. We therefore focused here on X-ray irradiation of 8-day-old male pupae of *S. litura* and determined that the appropriate sub-sterilizing dose is around 75 Gy. The sub-radiation dose for males was usually equal to the sterilization dose for females of this species, because fertile radiation females were thought to cause unpredictably significant population increase in the wild (Bloem et al., 1999). In our study, we discovered that even at the dose of 150 Gy employed in the experiment, the female could not be sterilized. Seth and Sehgal (1993) found that 100 Gy and 130 Gy doses of γ-ray irradiation were good sub-sterilisation doses of *S. litura*, but that the γ-ray of 130 Gy did not reach the sterilization dose of females, which is similar to our findings. That was because studies have showed that after 130 Gy of γ-ray irradiation, the male sperm competitiveness of *S. litura* decreased (Seth et al., 2002). Similar effects have been observed in other Lepidoptera insects. In *Spodoptera frugiperda*, males exposed to 100 Gy γ-ray dos exhibit better sperm competitiveness than males exposed to 150 Gy dose (Carpenter and Gross, 1993). As a result, if the female sterilization dose is used as the male sub-sterility dose, the competitive ability of *S. litura* may be seriously impacted. As a result, populations for genetic sex separation (GSS) from released insects should be developed before SIT technology can be used on a large scale, such as *C. capitata*, *Bombyx mori*, *P. gossypiella* (Peloquin et al., 2000; Tamura et al., 2000; Sahara et al., 2003; Augustinos et al., 2017).

Furthermore, flying ability is an essential factor to be concerned in the SIT program (Seth et al., 2016b). According to Stephens et al. (2006), the flight ability of sterile painted apple moths was not significantly affected after being irradiated with γ-rays at 100 Gy. The flight capacity of *Teia anortoides* was unaffected by γ-ray radiation of 160 Gy (Suckling et al., 2002). The flying ability of *S. frugiperda* was not affected when the dose of X-ray was lower than 300 Gy (Jiang et al., 2022). Their findings are nearly identical to ours.

Lepidopteran insects are more resistant to radiation than insects in other orders (LaChance et al., 1975) because, after irradiation, the shattered chromosomal fragments attach to the centromere and are passed to the next generation through germ cells (Proverbs and Newton, 1962; Marec and Traut, 1993). As a result, using substerile doses to irradiate insects can not only inhibit the development of parental populations but also reduce the number of their offspring (Seth et al., 1997; 2016a). This inherited sterility has provided remarkable advancement in the control of lepidopteran pests, such as *Trichoplusia ni* and *Helicoverpa zea* (North and Holt, 1969; Carpenter and Gross, 1993). The reproductive peak of F_1_ females was similar to that of normal insects, and there was no significant variation in the development duration of F_1_ generations in different stages by irradiation with the dose range of 25 Gy–75 Gy in this study. This shows that irradiated insects’ reproduction behavior can be synchronized with that of normal insects in the wild after being released in the field. The same effects were observed in research of *Helicoverpa Armigera* and *A. transitella* F_1_ generation developmental stages (Kim et al., 2015; Light et al., 2015). The F_1_ generation of irradiated insects in this study still showed high sterility (>60%) and the mating rate was unaffected, confirming that 75 Gy was a suitable dose of sterility. Similar results were reported when *S. litura* was irradiated with gamma rays, the substerilizing irradiation 0–24 h old male adults of *S. litura* with 100 Gy and 130 Gy of γ-ray used by Seth achieved a higher sterility rate for the F_1_ generation than for the parents, and 130 Gy also affected larval and gonadal development of the F_1_ generation (Seth et al., 2000; Seth and Sharma, 2001).

The goal of SIT is to inhibit wild populations from expanding by providing large populations of irradiated individuals to mate with wild populations. As a result, determining the appropriate release ratio of irradiated males to nonirradiated wild males so that a substantial number of irradiated males compete with the wild population to suppress the pest population is essential for the highest efficacy (Bloem and Carpenter, 2001; Bloem et al., 2005). The release ratio is considered as part of the SIT program. *Anopheles arabiensis* exhibits better mating competitiveness when three times more irradiated males were exposed to 70 Gy doses of γ-rays than normal males, according to Helinski and Knols (2009). The hatching rate of *A. albopictus* females was less than 20% when the ratio of irradiated males (irradiated by γ-rays of 35 Gy dose) to normal males was 10:1. In this study, the release rate of male insects was also investigated. When the irradiated male moths (exposed to a 75 Gy X-ray during the pupal stage) to normal male release ratios were 10:1, 11:1, and 12:1, the sterility rate was greater than 85%, this is comparable to the effectiveness of pesticides such profenofos and imidacloprid in controlling *S. litura* (Abbas et al., 2012; Abbas et al., 2014). In order to achieve a better control effect, we used logistic fitting to confirm that the rapid increase in sterility rate ended at a release ratio of 12.6:1, and that induced sterility rate might theoretically reach 91%. *S. litura* population growth will be severely hampered by this release ratio. An increase in the release rate is required to limit population expansion in many sterile insect release studies. After irradiating *Anastrepha fraterculus* with a 40 Gy X-ray dose, the ideal release ratio of irradiated male to normal male is 50:1 (Mastrangelo et al., 2018). The induced sterility rate can be increased to more than 70% by raising the release ratio of irradiated *C. capitata* male to normal male to 100:1 (Rendón et al., 2004). As mentioned earlier, with X-ray doses of 75 Gy, we theoretically determined the best release ratio to be 12.6:1 irradiated to nonirradiated males. However, because this ratio was determined in the laboratory, field testing is needed to determine the appropriate release ratio (Seth and Sehgal, 1993; Hofmeyr et al., 2005).

Using SIT alone cannot adequately control insects that migrate long distances, but the release of substerilized pests is compatible with other pest control methods (Knipling, 1964), and can be part of synergistic management that includes, for example, the release of substerilized pest insects with natural enemies of the pests (Seth et al., 2009; Cagnotti et al., 2016). Area-wide integrated pest management (AW-IPM), in conjunction with SIT, has also provided remarkable control of lepidopteran pests, using *Bacillus thuringiensis* to control *T. anartoides* (Suckling et al., 2007) and using Bt cotton to eradicate *P. gossypiella* (Saunders) in the continental United States and northern Mexico (Tabashnik et al., 2021). Such strategy also is good for the control of *S. litura*, which survives in winter season only in the tropical area of China (such as Hainan, Guangxi, Yunnan) (Fu et al., 2015; Bei et al., 2001). As a result, the activity range of *S. litura* is quite limited and isolated in the winter season, it should be a good option to manage the pest using the SIT strategy. The Bt corn planting is now legal in China (http://www.moa.gov.cn/ztzl/zjyqwgz/spxx/201912/t20191230_6334015.htm). By planting Bt corn in areas where *S. litura* overwinters and releasing sterile males in the winter in those areas, *S. litura* damage can be reduced there and nationwide as the moths migrate in the spring.

Although the radiation biology of *S. litura* was investigated in this study, there are still a number of issues to be resolved before the insect is released on a broad scale. Low-energy X-ray irradiators had a lower dose rate than γ-ray irradiators, and the relative efficiency of 30 kV–280 kV X-rays is about 8.9% lower than ^60^Co (Hjørringgaard, et al., 2020). This implies that X-rays might take a longer time to irradiate than ^60^Co. Current research indicated that the role of low-energy X-rays on insects was quite comparable to that of γ-rays, which could achieve high sterility (Yamada et al., 2014; Kumano et al., 2018). Recently, the X-ray irradiator had been upgraded, tweaked, and improved on a routine basis (Mastrangelo et al., 2010; Fan and Niemira, 2020). Furthermore, although there is a feasible feed for large-scale rearing of *S. litura* (Gupta et al., 2005), compared to easier rearing of dipteran insects, rearing *S. litura* on a large scale is expensive, so efficient, cheaper rearing methods need to be developed. Overall, X-ray irradiation technology to substerilize *S. litura* is safe and “green,” may play an important and special role compared to existing control methods to the pest. Therefore, there is a great necessary to take a field trial for establishing a mature method for deployment in a large-scale in southern China.

## Data Availability

The original contributions presented in the study are included in the article/supplementary material, further inquiries can be directed to the corresponding author.
